# The dissemination of C10 cysteine protease genes in *Bacteroides fragilis *by mobile genetic elements

**DOI:** 10.1186/1471-2180-10-122

**Published:** 2010-04-23

**Authors:** Roibeard F Thornton, Todd F Kagawa, Paul W O'Toole, Jakki C Cooney

**Affiliations:** 1 Department of Life Sciences, University of Limerick, Limerick, Ireland; 2 Materials and Surface Science Institute, University of Limerick, Limerick, Ireland; 3 Department of Microbiology, & Alimentary Pharmabiotic Centre, University College Cork, Cork, Ireland

## Abstract

**Background:**

The C10 family of cysteine proteases includes enzymes that contribute to the virulence of bacterial pathogens, such as SpeB in *Streptococcus pyogenes*. The presence of homologues of cysteine protease genes in human commensal organisms has not been examined. *Bacteroides fragilis *is a member of the dominant *Bacteroidetes *phylum of the human intestinal microbiota, and is a significant opportunistic pathogen.

**Results:**

Four homologues of the streptococcal virulence factor SpeB were identified in the *B. fragilis *genome. These four protease genes, two were directly contiguous to open reading frames predicted to encode staphostatin-like inhibitors, with which the protease genes were co-transcribed. Two of these protease genes are unique to *B. fragilis *638R and are associated with two large genomic insertions. Gene annotation indicated that one of these insertions was a conjugative Tn-like element and the other was a prophage-like element, which was shown to be capable of excision. Homologues of the *B. fragilis *C10 protease genes were present in a panel of clinical isolates, and in DNA extracted from normal human faecal microbiota.

**Conclusions:**

This study suggests a mechanism for the evolution and dissemination of an important class of protease in major members of the normal human microbiota.

## Background

*Bacteroides fragilis *is a Gram-negative member of the normal human gut microbiota. The *Bacteroidetes *constitutes one of the major bacterial phyla in the healthy human gut [1]. However, *B. fragilis *is also an important opportunistic pathogen, and it is the most frequently isolated anaerobic bacterium in clinical specimens, including abdominal abscesses and bloodstream infections [2]. Indeed, while *B. fragilis *accounts for only 4 to 13% of the normal human fecal microbiota, it is responsible for 63 to 80% of *Bacteroides *infections [3]. Only a few virulence factors have been described for *B. fragilis*, with the best characterized being the polysaccharide (PS) capsule [4] and a secreted metalloprotease, fragilysin [5]. The capsule, which displays antigenic variation, promotes the formation of abscesses [4], and the reduction of pro-inflammatory responses to *B. fragilis *[4,6]. The metalloprotease fragilysin, which has been linked to diarrheal disease [5], has activity against the zonula junctions between cells, and could disrupt tissue integrity [7]. *B. fragilis *also encodes homologues of C10 proteases [8]. These are members of the CA clan of papain-like proteases. Other C10 proteases include the important virulence factors Streptococcal pyrogenic exotoxin B (SpeB) from *Streptococcus pyogenes *and Interpain A from *Prevotella intermedia*. SpeB cleaves a variety of host protein, including immunoglobulin, fibronectin and vitronectin; it also activates IL-1β and releases kinin from kininogen [9]. Interestingly, both SpeB and Interpain A target and inactivate complement factor C3 [10,11]. One further characterized C10 protease is the Periodontain from the oral pathogen *Porphyromonas gingivalis*, which cleaves α1-proteinase inhibitor promoting degradation of connective tissue components [12].

For both SpeB and another well characterized family of cysteine proteases (C47 family) expressed in staphylococci (Staphopain), the protease genes are found juxtaposed to genes encoding specific protease inhibitors, Spi [13] (a propeptide analogue) and Staphostatin [14] (a lipocalin-like entity), respectively.

The genomes of *Bacteroides *spp., including *B. fragilis*, may include plasmids [15], and typically include multiple prophage remnants, pathogenicity islands and both conjugative and non-conjugative transposons (CTn and Tn respectively) [16]. This would facilitate acquisition and dissemination of virulence markers. Indeed, the fragilysin is encoded on a pathogenicity island which has been shown to be mobile [17].

This study centers on the identification and characterization of genes encoding homologues of SpeB, their genetic linkage with putative inhibitors, and the association of these homologous genes with mobile genetic elements.

## Results

### The *B. fragilis *genome harbours four paralogous C10 protease genes

**Figure 1:**
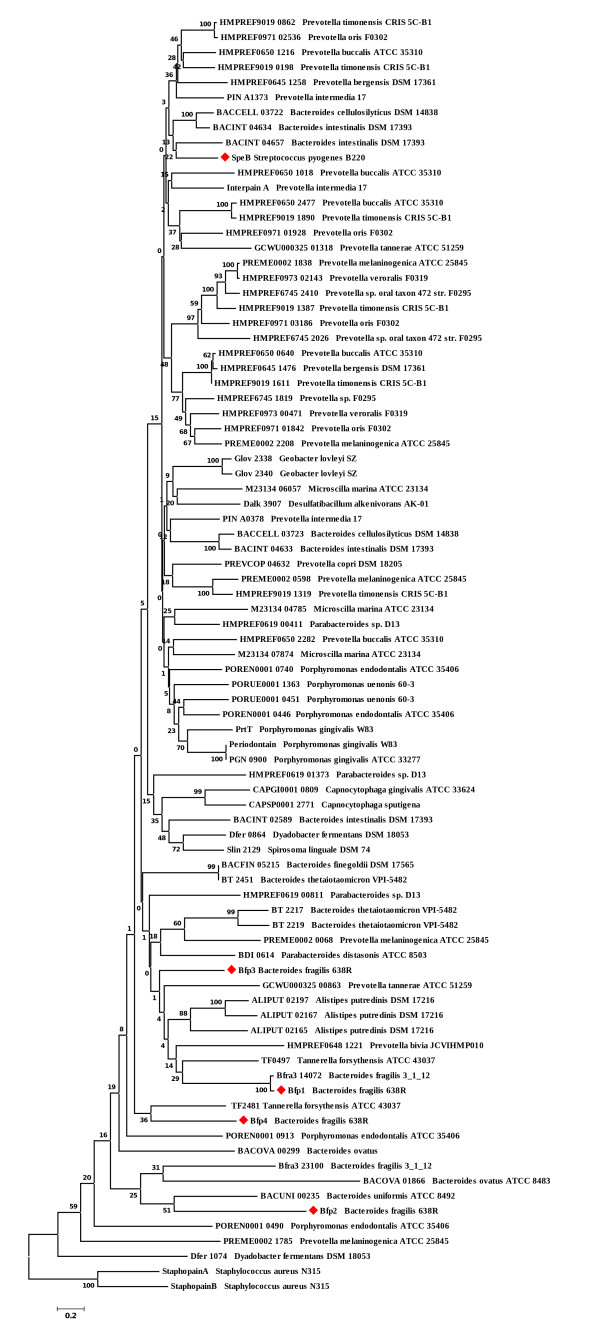
**Phylogenetic tree of the C10 proteases available on the GenBank and NCBI databases**. Cluster analysis was based upon the neighbour-joining method. Numbers at branch-points are percentages of 1000 bootstrap re-samplings that support the topology of the tree. The tree was rooted using C47 family cysteine protease sequences (Staphopains). The locus tag identifiers and the organism name are given. SpeB and the Btp proteases are indicated by a red diamond.

**Figure 2:**
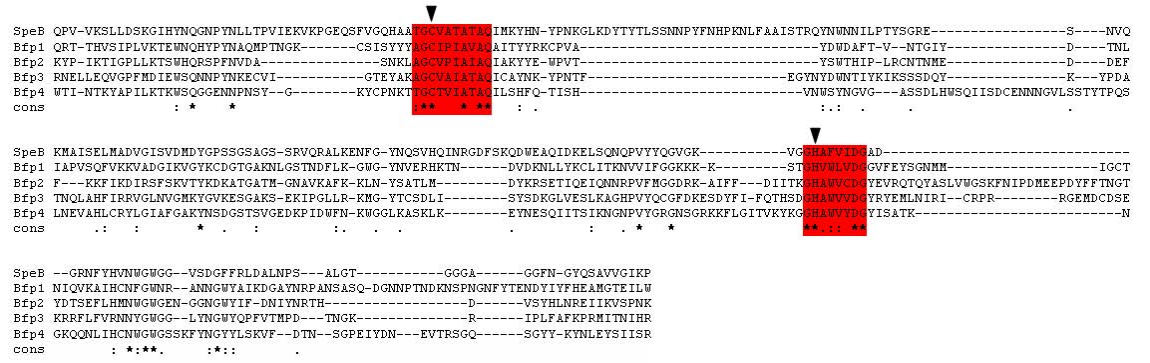
**Amino acid sequence alignment of the *Bacteroides fragilis *proteases Bfp with the archetype C10 protease SpeB from *Streptococcus pyogenes***. The alignment was generated with T-coffee [55]. The red back-highlight regions indicate the sequences flanking the critical active site Cys and His residues (vertical black arrowhead).

A phylogenetic study was undertaken to determine the relatedness of C10 proteases in other members of the *Bacteroidetes *phylum (Fig. 1). This identified eight-four C10 protease candidates, ranging in size from 269 to 1656 amino acids, in organisms that occupy both human and environmental niches. The larger of these proteins (>600 amino acid residues, average length 803 residues) group together along with SpeB and Interpain A. These larger proteins have additional C-terminal domains, the role of which is yet to be determined [12,18]. The Bfp proteases group with proteins <500 amino acid residues in length (average length 435 residues). Although acceptable bootstrap values were obtained for nodes separating deeper phylogenetic levels, the bootstrap values for the shallower divisions were low. This reflects the unstable phylogeny obtained. However, it is noteworthy that all of the candidate protease sequences had a variation on the two active site motifs indicated in Fig 2.

Of particular interest was the identification of SpeB homologues in *B. fragilis*. Analysis of the *B. fragilis *638R ftp://ftp.sanger.ac.uk/pub/pathogens/bf/, YCH46 [19] and NCTC9343 [7] genome sequences identified genes encoding a paralogous family of C10 cysteine proteases named Bfp1 (BF638R0104, 45390), Bfp2 (BF638R1641, 56666), Bfp3 (BF638R3679, 47323), Bfp4 (BF638R0223, 48433) for B. *fragilis *protease, encoded by genes *bfp1-4 *respectively. The locus identifiers for the unpublished 638R genome, followed by the predicted molecular mass of the preproprotein in Daltons are given in parenthesis. *bfp1 *and *bfp2 *were present in all three strains whereas *bfp3 *and *bfp4 *were present only in *B. fragilis *638R (Table 1).

**Table 1:** Occurrence of *bfp *genes in clinical isolates and in the human gut microbiota.

Strain	*bfp1*	*bfp2*	*bfp3*	*bfp4*	Bfgi2	*attB*
638R	**+**	**+**	**+**	**+**	**+**	**+**
YCH46^a^	**+**	**+**	**-**	**-**	**-**	**+**
NCTC9343^b^	**+**	**+**	**-**	**-**	**-**	**+**
NCTC9344	**+**	**+**	**+**	**-**	**+**	**+**
NCTC10581	**+**	**+**	**-**	**-**	**-**	**+**
NCTC10584	**-**	**+**	**-**	**-**	**-**	**+**
NCTC11295	**-**	**+**	**-**	**-**	**-**	**+**
NCTC11625	**+**	**+**	**-**	**-**	**-**	**+**
TMD1	**+**	**+**	**+**	**+**	**+**	**+**
TMD2	**+**	**+**	**+**	**+**	**+**	**+**
TMD3	**+**	**+**	**+**	**+**	**+**	**+**

^a. ^Based on analysis of genome sequence only, locus identifier BF0154 for *bfp1*, and BF1628 *bfp2*. All other strains confirmed by PCR.

^b. ^Locus identifier BF0116 for *bfp1 *and BF1640 for *bfp2*.

TMD1-TMD3: total microbiota DNA, from faeces of 3 healthy adult subjects.

Similarity between the predicted Bfp protein sequences and zymogen SpeB ranges from 33-41.2%, with similarity between the paralogues themselves higher (36.7-46.1%) (Table 2). These low values are not surprising, as it has been established that the overall sequence identity and similarity between the CA clan of Papain-like proteases is low [20]. However, the core of the the protease domains of the C10 proteases SpeB (1DKI) and Interpain (3BBA) [18] are similar in structure (root mean squared deviation of 1.220 Å based on 197 Cα positions), even with only 32.5% sequence identity. Critically, the active site residues (Cys165 and His313, SpeB zymogen numbering [21]) are highly conserved (Fig. 2). It is probable that the *bfp *genes encode active proteases, and thus, may contribute to the pathogenesis of *Bacteroides *infections in a manner analogous to the role of SpeB in streptococcal pathogenesis [22].

**Table 2:** Similarity/identity matrix for Bfp proteases and SpeB^a^.

C10 Protease	SpeB	Bfp1	Bfp2	Bfp3	Bfp4
SpeB		**19.2**	**22.6**	**16.7**	**21.9**
Bfp1	*38.1*		**21**	**23.9**	**19.7**
Bfp2	*33.0*	*36.7*		**20.2**	**22.5**
Bfp3	*41.2*	*41.7*	*37.7*		**28.5**
Bfp4	*38.2*	*42.1*	*41.0*	*46.1*	

^a ^Numbers in italics are percentage similarity, numbers in bold type are percentage identities.

**Figure 3:**
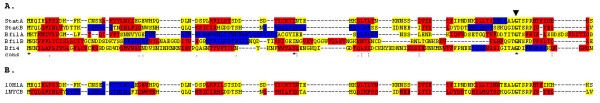
**Structure and sequence based alignments of Staphostatins with putative inhibitors from *Bacteroides fragilis***. Panel A is a sequence alignment generated with T-coffee. Superimposed on this are secondary structure predictions for all 5 proteins, generated with GorIV [46]. Residues with secondary structure assigned as coil, β-strand, and α-helix are back-highlighted in yellow, red and blue respectively. The glycine residue conserved in Staphostatins is marked with a vertical black arrowhead. Panel B is a sequence alignment of Staphostatin A (1OH1A [56]) and Staphostatin B (1NYCB [14]). The sequence based alignment was generated with T-coffee. This alignment is coloured, as for panel A, according to secondary structure determined from the crystal structures of the two inhibitors. For clarity the spacing is preserved from panel A. These alignments suggest that GorIV is over-predicting helical content in the staphostatins.

Bacterial cysteine protease genes have been found coupled to genes encoding specific inhibitors, therefore, the regions both up and downstream of the four *bfp *genes were analyzed for candidate inhibitors. Three open reading frames encoding small proteins (116-138 amino acids) within 35 base pairs of the proteases were identified. These were named *bfi1A *(BF638R0103), *bfi1B *(BF638R0105) and *bfi4 *(BF638R0222) (for *Bacteroides *f*ragilis*inhibitor). The encoded proteins showed no significant identity to the propeptides of any known protease, nor to Spi. Surprisingly, they had identity to the C47 cysteine proteases inhibitors, the Staphostatins, ranging from 15.0-23.4% identity and 32.6-45.7% similarity (Table 3). This is in line with identity between Staphostatin A and Staphostatin B with 20.4% identity and 45.0% similarity. Despite low levels of sequence identity, analysis of the predicted secondary structure and the conservation and alignment of a critical glycine residue in these sequences (indicated in Fig. 3) when compared to Staphostatins, suggested that these *bfi *genes encode specific protease inhibitors.

**Table 3:** Similarity/identity matrix for Bfi putative inhibitors, Staphostatins and Spi^a^.

	Spi	ScpA	SspB	Bfi1A	Bfi1B	Bfi4
Spi		**16.4**	**11.9**	**11.1**	**17.2**	**14.3**
ScpB^b^	*41.7*		**20.4**	**20.2**	**19.4**	**23.4**
SspC^b^	*31.2*	*45.0*		**20.2**	**18.6**	**15.0**
Bfi1A	*26.7*	*38.8*	*45.7*		**20.3**	**20.4**
Bfi1B	*35.7*	*39.7*	*40.5*	*41.3*		**20.1**
Bfi4	*31.2*	*39.1*	*32.6*	*38.4*	*39.9*	

^a ^Numbers in italics are percentage similarity, numbers in bold type are percentage identities.

^b ^ScpB and SspC are Staphostatin A and Staphostatin B respectively.

To determine the likely cellular location of Bfp and Bfi proteins, the respective sequences were analyzed using LipPred [23], LipoP [24], SignalP [25] and PSORTb [26]. These analyses suggested that Bfi1A has a typical Sec pathway leader sequence and is likely to be exported to the periplasm. Bfi1B, Bfi4, Bfp1, Bfp2 and Bfp4 have predicted lipoprotein signal sequences and are likely to be tethered to the outer membrane [24,27]. Whilst Bfp3 has a lipoprotein leader sequence it is not clear which membrane it is likely to associate with. It should be noted that maturation of C10 zymogens would release the active protease from the anchoring acyl-lipid into the extracellular milieu.

### *B. fragilis *C10 proteases genes, *bfp1 *and *bfp4*, are co-transcribed with those for predicted Staphostatin-like inhibitors

**Figure 4:**
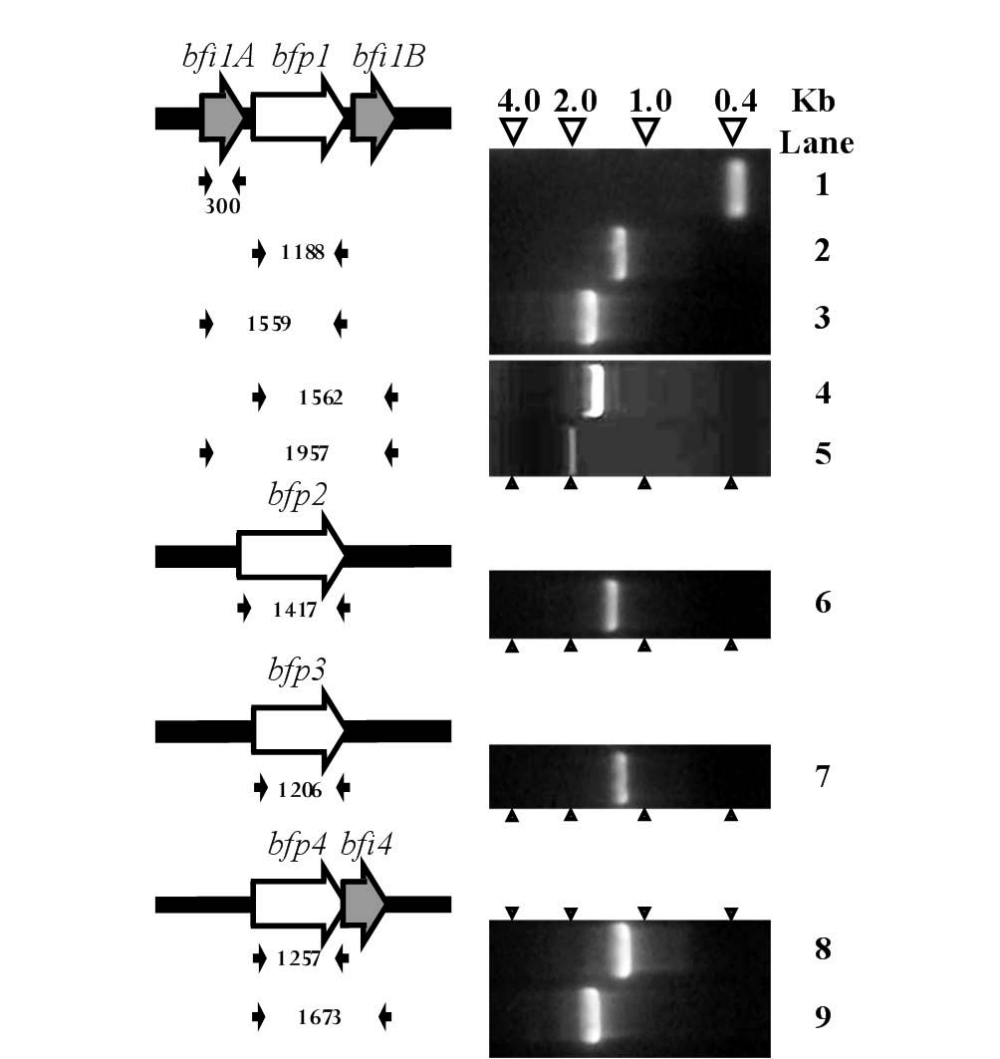
**Analysis of expression and transcriptional coupling of *bfp *genes in *Bacteroides fragilis***. Horizontal open arrows represent the protease (white) and putative inhibitor (grey) genes. Small filled black arrows represent the positions of the oligonucleotide primers used in the reverse-transcription PCR analysis, the size of the expected amplicon is given in bp between the appropriate sets of pimers. The resulting PCR fragments are presented in the right-hand panels, above which the size markers are indicated.

For both the streptococcal and staphylococcal systems, the proteases and adjacently encoded inhibitors are co-transcribed [13,28]. To determine if this transcriptional coupling of protease and inhibitor genes was also present in *B. fragilis*, RNA was isolated from broth grown 638R cells, and analysed by reverse transcriptase PCR, using a series of specific primers for the protease and inhibitor genes (Table 4). Amplicons were detected for all C10 protease structural genes suggesting that all the proteases were transcribed *in vitro *(Fig. 4, Lanes 2, 6, 7 and 8 for *bfp1*, *bfp2*, *bfp3 *and *bfp4 *respectively). Amplification of a 1.9 Kb product (Fig. 4, Lane 5) using primers Bfi1A_F and Bfi1B_R supports the hypothesis that *bfp1 *is co-transcribed on a single mRNA with *bfi1A *and *bfi1B*. In addition, amplification of a 1.65 Kb product with primers Bfp4_F and Bfi4_R suggests that *bfp4 *is transcriptionally coupled to *bfi4 *(Fig. 4, Lane 9).

**Table 4:** Oligonucleotide primers used in this study.

Primer	Sequence	Comment^a^
Bfp1_F	CAGCAGCATATGGACGAAGAAATCATTATTTTGATTAAT	E, L
Bfp1_R	CAGCAGGGATCCTTACCACAAAATTTCAGTTCCC	E, L
Bfp2_F	CAGCAGCATATGACAAGAAGAGTTGATTCTGCCAG	E
Bfp2_R	CAGCAGGGATCCTTATTTATTAGGTGACACTTTAAT	E
Bfp3_F	CAGCAGGGATCCAGAAGATAATGTAATTGCTTCTTT	E
Bfp3_R	CAGCCAGGAATTCTCATCGGTGTATATTGGTTATC	E
Bfp4_F	CAGCAGGGATCCGAAGACAATTTAGAATCTTTAA	E, L
Bfp4_R	CAGCAGGGATCCTCATCGCGATATAATAGAATATTC	E
Bfi1A_F	CAGCAGGAATTCGAGGATGTAATGGCTATTATG	E, L
Bfi1A_R	CAGCAGGGATCCTTACCTTCCAATATAAATGTC	E
Bfi1B_F	CAGCAGGGATCCACACCAACCAGATACTCCACC	E
Bfi1B_R	CAGCAGGAATTCTTACTCTTTTTTTTCGGCTGTG	E, L
Bfi4_F	CAGCAGGAATTCAGGGATGGAGATTGGGATTC	E
Bfi4_R	CAGCAGGGATCCTTAATTATCCTTTCCCTTTTGTTT	E, L
Bfgi2_Int_F	CCTGATATTAGCTTCTCTATCTTTTTTGCC	I
Bfgi2_Int_R	CAGCAGGGATTCCGAAGATAATGTAATTGCTTC	I
Bfgi2_attB_F	CCGGGAATGTTTCGTCAGGAATTGATGGTG	I
Bfgi2_attB_R	GGTTTATTGATTGTTATTTGTCGGCAAAG	I

^a ^Primer used in E = Expression studies, L = Linkage studies, I = Integration/Excision studies

### *bfp3 *and *bfp4 *are located on genome insertions

**Figure 5:**
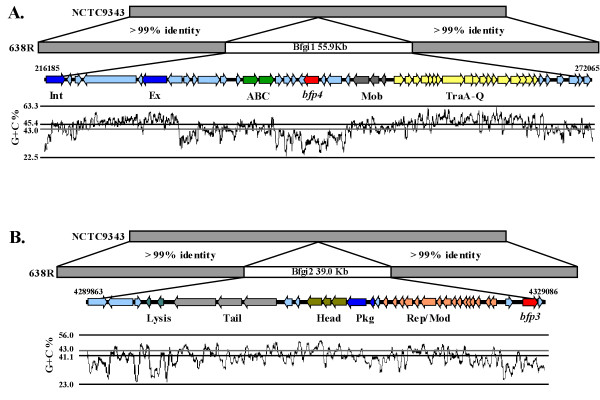
**Insertions in the genome of *Bacteroides fragilis *638R carry C10 protease homologues**. Genome alignment of *B. fragilis *strains 638R and NCTC9343 was generated using the Artemis Comparison Tool. The co-ordinates for the insertions are from the unpublished 638R genome. Genes in the insertions are represented by horizontal open coloured arrows and are described below (see also Tables 5 and 6). The G+C content of the insertions is plotted in the lowest section of each panel. The grey horizontal line in each case represents the average G+C content for the genome. For both panels the C10 proteases are represented by horizontal red arrows and the pale blue arrows are genes that are not directly related to the skeleton of the particular mobile genetic element. Panel A. The insertion Bfgi1 has the features of a CTn. The putative integrase and excisionase genes (Int and Ex respectively), ABC transporters (ABC), mobilization genes (Mob), and transfer genes (Tra) are represented by royal blue, dark green, grey and yellow arrows respectively. Panel B. The insertion Bfgi2 has the architecture of a Siphoviridae bacteriophage. The lysis cassette, tail region, head regions, packaging (Pkg) and the replication and modification genes (Rep/Mod) are represented by teal, mid-grey, moss green, royal blue and peach arrows respectively.

As mentioned above, two of the protease genes (*bfp3 *and *bfp4*) were identified only in strain 638R enabling a comparison with the two other sequenced strains of *B. fragilis*. Using the Artemis comparison tool [29], alignment of the *B. fragilis *NCTC9343 and *B. fragilis *638R genome sequences identified two large insertions in strain 638R associated with the chromosomal locations of *bfp3 *and *bfp4*. In *B. fragilis *638R, *bfp4 *was found on a 55.9 Kb insertion, called Bfgi2 in this study. Annotation of this insertion revealed an architecture similar to the CTnERL-type conjugative transposons (CTn) [30] (Fig. 5, panel A and Table 5). Although the expected integrase, excisionase and transfer regions were present in Bfgi1, mobility of this insertion could not be established for broth grown cultures treated with mitomycin C, tetracycline, or UV treatment (data not shown). These treatments are commonly used to initiate excision of CTn elements [31,32]. Bfgi1 showed homology to a region in *Porphyromonas gingivalis *ATCC 33277 which has previously been characterized as a CTn [33]. However, this region of ATCC 33277 did not encode a C10 protease.

**Table 5:** Annotation of genes in the *B. fragilis *638R Bfgi1 insertion.

ORF	Protein Length	Putative function	% Id/Sim^a^	Organism^b^	Accession no.^c^
1	411	Integrase protein	59/74 (411)	*B. fragilis *YCH46	AAS83518.1
2	119	Hypothetical protein	42/64 (114)	*B. thetaiotaomicron*	AA077037.1
3	162	Ctn042	37/59 (112)	*B. fragilis *YCH46	AAS83514.1
4	1828	DNA Methylase (BmhA)	57/71 (1339)	*B. fragilis *YCH46	AAS83508.1
5	143	Hypothetical protein	41/56 (121)	*B. thetaiotaomicron*	AA077432.1
6	709	Excisionase	57/72 (704)	*B. fragilis *YCH46	AAS83511.1
7	464	Hypothetical protein	41/57 (482)	*B. thetaiotaomicron*	AA075210.1
8	260	TetR/AcrR family	32/58 (204)	*B. thetaiotaomicron*	AA075614.1
9	161	Hypothetical protein	48/71 (108)	*P. gingivalis *W83	AA075614.1
10	780	Putative TonB OM Receptor	63/78 (780)	*B. fragilis *YCH46	BAD47377.1
11	412	Hypothetical protein	56/73 (398)	*B. fragilis *YCH46	CAH06331.1
12	187	Putative Ni-Co-Cd resistance protein	29/42 (110)	*Syntrophus aciditrophicus *SB	ABC78121.1
13	604	ABC Transporter	41/61 (570)	*B. thetaiotaomicron*	AA075616.1
14	593	ABC Transporter	43/63 (591)	*B. thetaiotaomicron*	AA075615.1
15	172	RteC	56/76 (80)	*B. thetaiotaomicron*	AAA22922.1
16	129	Peptidase S51	44/59 (100)	*Listeria monocytogenes*	AAT03167.1
17	114	Hypothetical protein	69/79 (73)	*P. gingivalis *W83	AAQ66123.1
18	138	Hypothetical protein	34/53 (135)	*B. thetaiotaomicron*	AA077558.1
19	431	C10 protease	26/43 (454)	*B. thetaiotaomicron*	AA077558.1
20	112	Hypothetical protein	27/72 (80)	*Polaribacter irgensii*	A4BZ61
21	512	ECF type σ-factor	31/50 (502)	*B. thetaiotaomicron*	AA077884.1
22	148	Hypothetical protein	43/58 (46)	*Campylobacter upsaliensis*	EAL52724.1
23	671	MobC	51/91 (660)	*B. fragilis *YCH46	AAS83500.1
24	408	MobB	53/71 (348)	*B. fragilis *YCH46	AAS83499.1
25	137	MobA	46/66 (136)	*B. fragilis *YCH46	AAS83498.1
26	260	TraA	53/71 (246)	*B. fragilis *YCH46	AAG17826.1
27	142	TraB	34/51 (133)	*B. fragilis *YCH46	BAD48110.1
28	135	TraC	34/55 (63)	*B. fragilis *YCH46	AAS83495.1
29	271	TraA	37/53 (251)	*B. fragilis *YCH46	BAD49765.1
30	196	TraD	26/37 (182)	*B. thetaiotaomicron*	AA077408.1
31	123	TraE	73/79 (78)	*B. fragilis *YCH46	BAD48110.1
32	126	TraF	56/66 (87)	*B. fragilis *YCH46	AAS83492.1
33	828	TraG	72/83 (829)	*B. fragilis *YCH46	BAD466872.1
34	209	TraI	65/80 (209)	*B. fragilis *YCH46	BAD46870.1
35	366	TraJ	70/86 (303)	*B. fragilis *YCH46	AAS83488.1
36	207	TraK	75/84 (207)	*B. fragilis *YCH46	AAS83487.1
37	110	TraL	37/58 (72)	*B. fragilis *YCH46	BAD48102.1
38	454	TraM	49/64 (439)	*B. fragilis *YCH46	BAD46866.1
39	310	TraN	70/84 (300)	*B. fragilis *YCH46	AAG17839.1
40	194	TraO	55/72 (177)	*B. fragilis *YCH46	BAD46864.1
41	292	TraP	52/67 (292)	*B. fragilis *YCH46	BAD46863.1
42	153	TraQ	60/76 (139)	*B. fragilis *YCH46	BAD48097.1
43	171	Lysozyme	53/73 (147)	*B. fragilis *YCH46	BAD46861.1
44	116	DNA Binding protein	75/80 (103)	*P. gingivalis *W83	AAQ66295.1
45	530	Hemerythrin	41/62 (508)	*Alkaliphilus metalliredigens*	EA081668.1
46	426	Ctn003	41/57 (441)	*B. fragilis *YCH46	BAD46856.1
47	176	Anti-restriction protein	52/71 (175)	*B. fragilis *YCH46	BAD48093.1
48	138	Ctn002	48/62 (115)	*B. fragilis *YCH46	BAD46855.1
49	200	Hypothetical protein	74/77 (31)	*B. fragilis *YCH46	BAD48092.1

^a ^Percentage identity/similarity, the number in parenthesis is the number of amino acids used in the calculations.

^b ^The organism encoding the *B. fragilis *638R gene homologue.

^c^Accession number of the highest scoring BLAST hit with an annotated function.

The *bfp3 *gene was located on a 39 Kb insertion, called Bfgi2 in this study. Analysis of this region predicted functional modules, e.g. DNA metabolism, DNA packaging, prophage head, tail and lysis proteins, consistent with a bacteriophage genomic structure similar to the Siphoviridae family of bacteriophages (Fig. 5, panel B and Table 6). These phage are known to infect bacteria that reside in the gut, and are the most frequently identified phage infecting *B. fragilis *[34]. Similarly to other Siphoviridae, Bfgi2 inserts into the 3' end of the tRNA^Arg ^gene [31]. The *attB *site overlaps the tRNA^Arg ^gene, however integration of Bfgi2 regenerates a functional tRNA^Arg ^gene. Bfgi2 had homology only with a region of a genome for an unidentified *Bacteroides *sp. (Bacteroides sp. 3_2_5), which included a homologue of *bfp3*.

**Table 6:** Annotation of genes in the *B. fragilis *638R Bfgi2 insertion.

ORF	Protein Length	Putative function	% Id/Sim^a^	*Organism *(Bacteriophage)^b^	Accession no.^c^
1	446	Integrase	47/63 (436)	*Bacteroides uniformis*	AAF74437.1
2	751	Polysialic acid transport protein, KpsD	72/84 (676)	*B. fragilis *YCH46	BAD48680.1
3	163	Hypothetical protein	37/49 (156)	*B. fragilis *YCH46	BAD49193.1
4	172	N-acetylmuramyl-L-alanine amidase	60/75 (150)	*B. thetaiotaomicron*	AA077433.1
5	151	Holin	25/54 (99)	*B. subtillus *(phi-105)	NP_690778.1
6	1215	Phage related protein, tail component	26/49 (173)	*Actinobacillus pleuropneumonia*	ZP_00134779.1
7	697	Hypothetical protein	21/40 (300)	*Flavobacterium *(11b)	YP_112519.1
8	1034	Tail tape measure protein	31/50 (119)	*Burkholderia cepacia *(BcepNazgul)	NP_918983.1
9	195	Hypothetical protein	32/54 (150)	*B. fragilis *YCH46	BAD49201.1
10	126	Hypothetical protein	29/52 (86)	*B. fragilis *YCH46	BAD49202.1
11	425	Phage major capsid	32/50 (252)	*Vibrio *phage VP882	AAS38503.2
12	204	Prohead protease	42/59 (157)	*Lactobacillus casei *(A2)	CAD43895.1
13	450	Phage portal protein	34/52 (365)	*Pseudomonas *(D3)	AAD38955.1
14	543	Terminase (Large subunit)	38/58 (493)	*Streptococcus agalactiae *(λSa04)	ABA45667.1
15	145	Terminase (Small subunit)	26/43 (122)	*Lactococcus lactis *(Bil309)	NP_076733.1
16	139	Hypothetical protein	28/59 (171)	*Clostridium difficile 630*	CAJ67750.1
17	104	HNH Endonuclease	41/59 (74)	*Geobacillus *(GBSVI)	ABC61271.1
18	142	Hypothetical protein	98/100 (136)	*B. fragilis *YCH46	BAD49213.1
19	104	Hypothetical protein	97/100 (93)	*B. fragilis *YCH46	BAD49214.1
20	320	Hypothetical protein	99/100 (294)	*B. fragilis *YCH46	BAD49215.1
21	113	Hypothetical protein	99/99 (109)	*B. fragilis *YCH46	BAD49216.1
22	428	Ctn003	39/53 (420)	*B. fragilis *YCH46	AAS83476.1
23	175	Ctn002	35/48 (134)	*B. fragilis *YCH46	AA583475.1
24 25	253 137	Putative DNA Methylase	100/100 (253)	*Lactococcus lactis *(Tuc2009)	NP_108695.1
26	124	Hypothetical protein	88/88 (116)	*B. fragilis *YCH46	BAD49220.1
27	150	NinG recombination protein	98/98 (125)	*A. actinomycetemcomitans *(AaPhi23) *bacteriophage *bb *bacteriophage*	NP_852744.1
28	126	Hypothetical protein	93/94 (116)	*B. fragilis *YCH46	YP_099756.1
29	149	DNA Topoisomerase I	32/51 (82)	*Pediococcus pentosaceus ATCC25745*	YP_80446.1
30	106	Excisionase	42/61 (52)	*Colwellia psychrerythraea 34H*	YP_268668.1
31	198	Hypothetical protein	66/74 (110)	*B. fragilis *YCH46	BAD49224.1
32	137	Peptidase S24	29/50 (81)	*Flavobacterium johnsoniae*	EASS8507.1
33	121	Hypothetical protein	35/52 (120)	*Pelobacter carbinolicus*	YP_358455.1
34	431	C10 protease	28/45 (375)	*B. thetaiotaomicron*	NP_811364.1

^a ^Percentage identity/similarity, the number in parenthesis is the number of amino acids used in the calculations.

^b ^The organism, with associated bacteriophage in parenthesis where applicable.

^c^Accession number of the highest scoring BLAST hit with an annotated function.

The regions flanking the C10 loci in a range of *Bacteroidetes *(*B. thetaiotaomicron *(AE015928), *B. uniformis *(AAYH00000000), *B. ovatus *(AAXF00000000), *B. intestinalis *(ABJL00000000), *Parabacteroides distasonis *(CP000140), *Porphyromonas gingivalis *(AP009380, AE015924) and *Prevotella intermedia *(ID: 246198) were examined for the presence of markers for mobile genetic elements (*e.g*. the Tra functional module, or phage structural modules for instance tail, and capsid). The GenBank accession code or JCVI taxon numbers are given in parenthesis. A cassette of Tra genes (A through O, locus tags PG1473-1486) was found 35.3 Kb away from *prtT *in *Porphyromonas gingivalis *strain W83 (locus tag 1427) and again in strain ATCC 33277 Tra I to Q were found (locus tags PGN_592 to PGN_599) 40.5 Kb away from PrtT (PGN_0561) in that strain. However, no complete CTn or phage could be found adjacent to these or any other C10 protease gene.

### The Bfgi2 element harbouring the *bfp3 *gene is capable of excision

**Figure 6:**
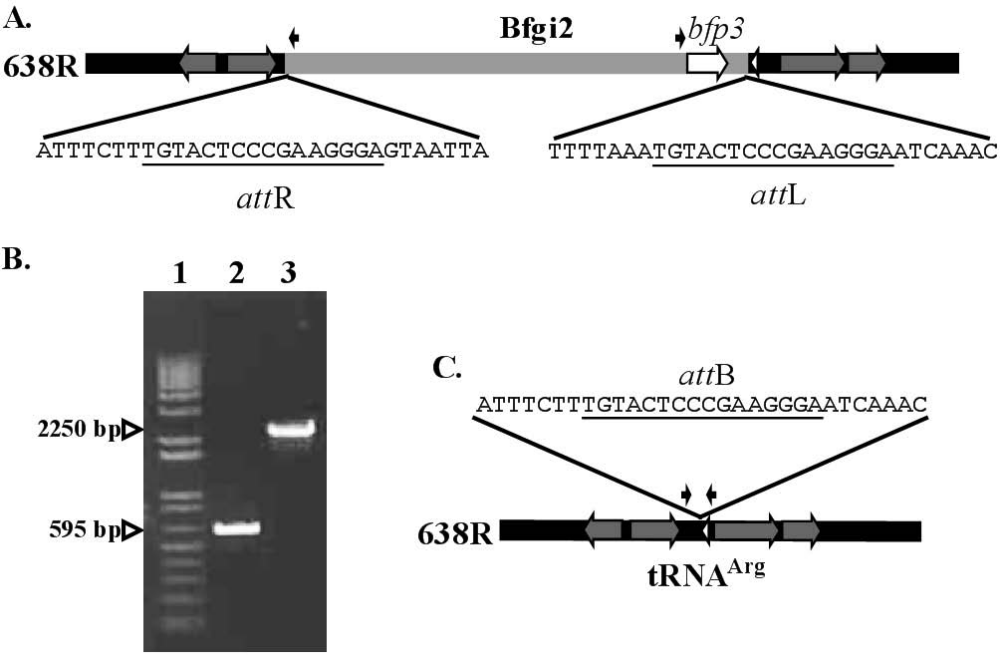
**The prophage carrying *bfp3 *is capable of excision**. Panel A. The Bfgi2 prophage (grey bar) is flanked by the *B. fragilis *638R genome (black bar). The *bfp3 *gene (open white arrow), *tRNA*^*Arg *^(white arrowhead) and genes flanking Bfgi2 (mid-grey) are shown. The *attR *and *attL *sequences (underlined) are shown in the expanded sequence. The locations of primers used in these studies are shown by small black arrows (see Table 4). Panel B. Agarose gel electrophoretic analysis of PCR reactions to test for excision of the prophage (Lane 2) and for the circular intermediate of the 'phage (Lane 3). Lane 1 contains DNA size markers. Panel C. Schematic representation of the 638R genome, after excision of the Bfgi2 element. Colour scheme is as for panel A. The regenerated *attB *site (underlined) is shown in the expanded sequence.

The putative *att *sequence for the integration of Bfgi2 was identified by analysis of the sequence at the boundaries of the inserted DNA in strain 638R compared with NCTC9343. A short 16 bp direct repeat sequence was identified flanking the Bfgi2 insertion (Fig. 6, panel A). PCR primers Bfgi2_attB_F and Bfgi2_attB_R (Table 4) were used in a PCR reaction to detect the excision of the Bfgi2 prophage from mitomycin C treated *B. fragilis *638R cells. The resulting 595 bp PCR product is consistent with excision of Bfgi2 from the *B. fragilis *638R genome (Fig. 6, panel B, Lane 2), and reconstruction of an intact tRNA^Arg ^gene (Fig. 6, panel C). Sequencing of this PCR product indicated the presence of a single copy of the 16 bp repeat region, the proposed *attB *site for Bfgi2 (Fig. 6, panel C).

The mitomycin C-treated cells were also analysed for the presence of the Bfgi2 circular intermediate. The primers Bfgi2_Int_F and Bfgi2_Int_R (Table 4) were designed directed outwards across the proposed *attL *and *attR *sites. Using these primers, amplification of product should only occur if a circularized form of Bfgi2 is present in the cell. The size (2.25 Kb) sequence of the resulting PCR product confirmed the presence of the circular intermediate (Fig. 6 panel B, Lane 3). Attempts to show plaque formation using NCTC9343 as an indicator strain did not produce any visible plaques. This could be due to the phenomenon of limited host range for the bacteriophage. However, given that Bfgi2 circular intermediate was detected it is tempting to speculate that it is, or is a derivative of an active phage and such phage could be transmitted to a non-lysogenized strain of *B. fragilis*, bringing with it a copy of a C10 protease.

### C10 protease genes are present in clinical isolates of *B. fragilis *and in the healthy human faecal microbiota

In addition to the 3 genome strains, a panel of 5 clinical isolates of *B. fragilis *from several human infection sites (Table 7) were tested by allele-specific PCR for the C10 protease genes they harbour. The results indicated that this panel of strains have a complement of *bfp *genes more similar to NCTC9343 than to 638R (Table 1). The distribution of *bfp *genes in the clinical isolates is not identical, and none of the 5 isolates carried all four *bfp *genes. The *bfp1-4 *genes were detected in 3, 5, 1 and 0 clinical isolates respectively. The *bfp4 *gene was not be detected in any of these clinical strains, while *bfp1 *was not detected in two strains (NCTC 10584 and NCTC 11295). In contrast, *bfp2 *was encoded by all strains. In *B. fragilis *strain YCH46, there is a CTnERL-type conjugative transposon 353 bp distance from the *bfi1A-bfp1-bfi1B *gene cluster. However, this conjugative transposon is not present in either of the other two sequenced *B. fragilis *genomes, 638R and NCTC 9343. The *bfp3 *gene was only detected in one clinical isolate (NCTC 9344), with a concomitant detection of the Bfgi2 insertion. In all cases a 595 bp fragment was successfully amplified using the primer pair Bfgi2_attB_F and Bfgi2_attB_R (not shown), indicating the presence of a free integration site for Bfgi2 in all strains. It should be noted that for NCTC 9344 and 638R, there was a lower product yield and although not quantitative this is likely due to the integration of Bfgi2 in a sub-population of the cells.

**Table 7:** Bacterial strains used in this study

*B. fragilis *strain	Source of isolate	Reference
638R	Clinical isolate, human	[57]
YCH46^a^	Bacteraemia, human	[19]
NCTC9343	Appendix abscess, human	[58]
NCTC9344	Septic operation wound, human	[59]
NCTC10581	Empyema fluid, human	[60]
NCTC10584	Pus, human	[58]
NCTC11295	Pus from fistula, human	[61]
NCTC11625	Post-operative wound infection, human	[62]

^a. ^Analysis of genome sequence only.

Presence of *bfp *genes in the healthy human intestinal microbiota was investigated by PCR analysis performed on total DNA extracted from faeces from three adult subjects. The amplification of the appropriately sized DNA fragments indicated that all 4 *bfp *genes characterized in this study were present in all three subjects whose samples were tested (Table 1). Interestingly, this analysis also indicated the presence of an integrated Bfgi2 prophage in these faecal samples, as well as free *attB *sites.

## Discussion

This study has established the presence of homologues of the streptococcal virulence factor SpeB in a significant gut microorganism, *B. fragilis*. The amplification of *bfp1-4 *specific sequences from mRNA samples supports the idea that these protease genes are expressed *in vivo *and in two cases the protease genes (*bfp1 *and *bfp4*) are coupled to genes encoding proteins resembling Staphostatins-like inhibitors. A role in protection of the bacterial cells from ectopic protease has been mooted for these inhibitors [35]. From sequence analysis, the Bacteroides inhibitors are likely to localize to the periplasm and cell membranes, which could be an additional mechanism to protect the bacterial cell from proteolytic damage, similar to roles suggested for Spi and the Staphostatins.

The presence of two Bfp protease genes on mobile genetic elements parallels some of the paradigms for the acquisition of virulence determinants by other microorganisms. For example the Panton-Valentine Leukocidin of *Staphylococcus aureus *[36], SpeC of *S. pyogenes *[37], diphtheria toxin of *Corynebacterium diphtheria *[38] and cholera toxin of *Vibrio cholera *[39] as well as the fragilysin of *B. fragilis *[40] are all encoded by mobile genetic elements. Although the latter case has yet to be conclusively established, the other examples cited, and many others in the literature, illustrate an augmentation of virulence in the recipient organism. Thus, the acquisition of additional copies of a protease with homology to SpeB by lateral gene transfer may increase the ability of *B. fragilis *to cause disease. However, establishing the mechanism of transfer of these protease genes and the role of the encoded proteases in *B. fragilis *opportunistic infections will require further studies.

## Conclusion

The phylum *Bacteroidetes *constitutes a major proportion of the healthy human intestinal microbiota. Variations in the *Bacteroidetes *proportion are linked to disease, and selected species are significant causes of human infectious disease. Alterations in the composition or function of the *Bacteroidetes *component of the intestinal microbiota might plausibly be involved in diseases involving immune dysregulation, including Inflammatory Bowel Disease, or Irritable Bowel Syndrome. Bacterial proteases are particularly relevant in this context, because they might be involved in the perturbed regulation of host matrix metalloproteases, which is a feature of IBD [41]. Thus the linkage of C10 proteases genes to mobile genetic elements in *B. fragilis*, and the demonstrated presence of these coding sequences in the healthy adult gut microbiota, is potentially significant. Experiments to investigate the expression and function of these genes *in vivo *are in progress.

## Methods

### Bacterial strains and culture conditions

*Bacteroides fragilis *strains used in this study are presented in Table 7. All strains were purchased from the United Kingdom National Culture Collection (UKNCC) except 638R which was a kind gift from Dr Sheila Patrick, Queen's University, Belfast. Both *B. fragilis *strains and *B. thetaiotaomicron *VPI-5482 [42] were grown in an anaerobic chamber at 37°C. Cultures were grown without shaking in Brain Heart Infusion (BHI) broth supplemented with 50 μg/ml hemin and 0.5 μg/ml menadione. Media for plating was made from Brain Heart Infusion agar supplemented with 5% defibrinated sheep blood, 50 μg/ml hemin and 0.5 μg/ml menadione.

### Bioinformatics and sequence analysis

Members of the C10 protease family in *B. fragilis *were detected by BLAST analysis [43]. Sequences were aligned by CLUSTAL W [44] or T-Coffee [45]. Protein secondary structure was predicted using GorIV [46] and JPred [47]. Protein export signals were identified using the algorithms using LipPred [23], LipoP [48], SignalP [25] and PSORTb [26]. Phylogenetic and molecular evolutionary analyses were conducted using genetic-distance-based neighbour-joining algorithms [49] within MEGA Version 4.0 http://www.megasoftware.net/. Bootstrap analysis for 1000 replicates was performed to estimate the confidence of tree topology [50]. MegaBLAST [51] was used to search all NCBI genomes for Bfgi1 and Bfgi2.

### Molecular techniques

Standard techniques were employed for molecular analysis [52]. *Bacteroides *genomic DNA was prepared as described by [53]. Total microbial DNA was extracted from human faeces, collected under an ethically approved protocol, by a glass beads-Qiagen Stool kit method previously described [54]. PCR reactions were carried using 10-30 ng of genomic DNA from *B. fragilis *638R as template and using Phusion Polymerase (New England Biolabs). The primers Bfp3_F and Bfgi2_Int_F (Table 4) were used for detecting the *attP *sites for Bfgi2.

Bfgi2_attB_F and Bfgi2_attB_R (Table 4) were used for determining the *attB *attachment sites for Bfgi2 integration. The primers TraQ_F and Int_F were used in testing for the presence of the circular intermediate for Bfgi1. Primers to detect the circular intermediate for both Bfgi1 and Bfgi2 were designed, pointing outwards, flanking the ends of each predicted element. Primers to detect the *attB *site in Bfgi2 were designed, pointing inwards, flanking the proposed excision point for the Bfgi2 prophage DNA.

### Total RNA isolation for Reverse Transcription analysis

*B. fragilis *638R and *B. thetaiotaomicron *VPI-5482 were cultured under anaerobic conditions until early logarithmic phase and the cultures were then immediately centrifuged for 15 minutes at 4000 × g. Total RNA extraction from *B. fragilis *638R and *B. thetaiotaomicron *VPI-5482 was carried out using the FastRNA^® ^Pro Blue Kit according to manufacturer's instructions (Q-Biogene, UK). Total RNA was subjected to DNase treatment using Turbo DNase (Ambion, UK) and stored at -80°C. RNA integrity was analyzed visually using denaturing 1.2% agarose gel electrophoresis and quantified using a NanoDrop (Thermo Fisher Scientific, USA). Reverse transcription PCR for C10 proteases was performed using the Superscript III One-step RT-PCR system (Invitrogen, USA). Primers used in RT-PCR reactions are documented in Table 4. Primers were added to a final concentration of 200 nM and 200 ng of total RNA added. As a control for DNA contamination, RT-PCR minus reactions was set up where the control reaction only received primers after the reverse transcriptase step. Aliquots (20 μl from 25 μl) of all samples were analyzed by standard agarose gel electrophoresis.

### Induction of Bfgi1 and Bfgi2 excision from the *B. fragilis *638R genome

*B. fragilis *638R was grown overnight and then sub-cultured by a 1 in 50 dilution into fresh broth and grown until late log phase. The culture was then exposed to either Mitomycin C (0.2 μg/ml), Tetracycline (0.5 μg/ml) UV light (1 mJ/cm^2^) then grown for a further 12 hours.

## Authors' contributions

RFT performed and designed experiments, and co-wrote the manuscript. TFK designed experiments and interpreted the data. PWOT designed experiments, analyzed data and co-wrote the manuscript. JCC conceived the study, designed the experiments, interpreted the data and co-wrote the manuscript. All authors read and approved the final manuscript.
